# A Pulsatile Flow-Modulation Microfluidic Sensor for Simultaneous Monitoring of Red Blood Cell Aggregation and Viscosity-Sensitive Time Constant

**DOI:** 10.3390/s26144541

**Published:** 2026-07-17

**Authors:** Yang Jun Kang

**Affiliations:** Department of Mechanical Engineering, Chosun University, 10, Chosundae 1-gil, Dong-gu, Gwangju 61452, Republic of Korea; yjkang2011@chosun.ac.kr; Tel.: +82-62-230-7052; Fax: +82-62-230-7055

**Keywords:** red blood cell aggregation, viscosity-sensitive time constant, microfluidic bifurcation, continuous pulsatile flow profile

## Abstract

**Highlights:**

**What are the main findings?**

- The proposed single-syringe-pump microfluidic method enables simultaneous measurement of the viscosity-related time constant (λ_1_) and RBC aggregation index (AI) under continuous pulsatile blood delivery, with λ_1_ showing a strong correlation with fluid viscosity.
- The optimized pulsatile-flow profile provides stable and reproducible measurements, while the proposed AI shows trends consistent with conventional aggregation indices and successfully detects time-dependent blood property changes during continuous infusion.

**What are the implications of the main findings?**

- The proposed method can simplify hemorheological sensing by replacing multi-pump or flow-stoppage-based measurements with a single-pump, continuous pulsatile-flow platform, making it more suitable for compact and practical blood monitoring devices.
- The ability to detect simultaneous changes in viscosity-related flow resistance and RBC aggregation index during continuous blood infusion suggests that this approach could be useful for real-time monitoring of blood sample variation, microfluidic diagnostics, and future point-of-care hemorheological assessment.

**Abstract:**

Red blood cell (RBC) aggregation and viscosity-related flow resistance are important hemorheological parameters for assessing blood flow abnormalities, but their simultaneous measurement often requires multiple pumps or intermittent flow stoppage. In this study, we propose a single syringe pump microfluidic sensing method for simultaneous evaluation of RBC aggregation and transient flow response under continuous pulsatile blood delivery. The device consists of a single inlet, a main straight channel, a bifurcated test channel, and a big outlet. An optimized pulsatile-flow profile was applied by periodically switching the flow rate between high flow rate (*Q*_h_ = 6 mL/h for 2 min) and low flow rate (*Q*_l_ = 1 mL/h for 4 min), and the transient velocity response was analyzed to extract the time constant (λ_1_) as a viscosity-related indicator. After optimization, the selected flow profile provides stable and reproducible measurements of both λ_1_ and the RBC aggregation index (AI) while reducing unnecessary blood consumption. The λ_1_ shows a strong correlation with viscosity and is significantly affected by syringe air compliance. The proposed AI exhibits consistent trends when compared with conventional aggregation indices. Furthermore, it exhibits temporal stability under continuous blood flow. Finally, the method is adopted to detect time-dependent changes in blood during continuous blood infusion, which demonstrates its potential as a simple, sensitive, and practical microfluidic sensor for real-time hemorheological monitoring.

## 1. Introduction

Red blood cell (RBC) aggregation is regarded as a key hemorheological phenomenon that has a strong impact on blood viscosity and fluidic resistance, thereby potentially influencing tissue perfusion under low shear rates [1,2,3,4]. Because RBC aggregation modulates microvascular flow resistance and oxygen transport, its quantitative assessment is clinically important for evaluating hemorheological alterations associated with inflammation, cardiovascular and metabolic disorders, and hematological diseases [5,6,7,8]. In addition, RBC aggregation is governed by multiple hematological factors, including plasma protein concentration, hematocrit, RBC morphology [9], cellular deformability [10], and membrane viscoelastic properties [3,6,11]. Therefore, reliable RBC aggregation assessment requires quantifying aggregation while considering key modulatory blood conditions, particularly hematocrit and plasma composition, which can affect disaggregation shear rate determination and aggregation measurements [12,13,14].

In conventional RBC aggregation assays, test blood is loaded into a measurement chamber and subjected to a high-shear field for a sufficient period to disrupt pre-existing aggregates. The external driving source, such as a vacuum pump [15], syringe pump [13], solenoid valve [16], or vibrational motor [17,18], is then abruptly turned off to stop blood flow, allowing RBC re-aggregation to occur under near-stagnant or low-shear conditions. The resulting aggregation process is subsequently monitored to determine aggregation kinetics or aggregation indices [19]. That is, several detection methods, including optical methods (LED or photodiode) [18], electrical impedance [20,21], ultrasound [14,22], and microscopic imaging [23,24], are adopted to quantify RBC aggregation. The RBC aggregation index (AI) is subsequently determined from the temporal syllectogram recorded during RBC re-aggregation. Although the previous methods can provide quantitative aggregation indices, they are often limited when the hematocrit or suspending medium changes under continuous flow or circulation conditions [13].

More recently, to resolve the critical issues raised by the previous methods, continuous flow-dependent RBC aggregation has been assessed using microfluidic bifurcation channels [13]. Herein, a single blood flow is divided into low-flow and high-flow branches, where RBC aggregation is promoted in the low-flow channel and dispersed in the high-flow channel. The aggregation index is calculated from time-lapse image intensities in both channels. Considering that hematocrit or plasma medium can be varied under continuous blood delivery [25,26,27,28], the previous method has been further improved to simultaneously measure blood viscosity and RBC aggregation [29]. However, the previous method relies on two precision pumps to introduce the reference fluid and the test blood sample simultaneously. Continuous infusion of 1× PBS can gradually dilute the test blood, making it difficult to distinguish true RBC alterations from hemodilution-induced changes during continuous blood flow [30,31,32]. To overcome this limitation, direct viscosity measurement using a reference fluid should be replaced by indirect viscosity-sensitive parameters [33,34].

In this study, we propose a microfluidic sensing method for simultaneous assessment of RBC aggregation and a viscosity-sensitive time constant under continuous pulsatile blood flow. The proposed device is designed to generate two distinct flow environments: a low-flow test chamber for inducing and measuring RBC aggregation and a high-flow main channel for analyzing transient velocity responses. A single syringe pump is used to generate a pulsatile blood-flow profile. In particular, in the low-flow test chamber, RBC aggregation is quantified using a position-dependent aggregation index derived from image intensity along the test chamber. This approach allows spatially resolved evaluation of RBC aggregation. In the high-flow main channel, the time-dependent velocity of the blood flow is measured and analyzed by regression to obtain a viscosity-sensitive time constant, which reflects changes in the state of blood delivered from the syringe.

Compared with previous methods that measure RBC aggregation alone [35], estimate viscosity using co-flow interface tracking [29,30], or perform sequential pressure or aggregation analysis, the proposed method enables simultaneous assessment of RBC aggregation and a viscosity-sensitive time constant in a single microfluidic platform under continuous pulsatile blood flow. By combining position-dependent image-based aggregation analysis in a low-flow test chamber with velocity-based regression analysis in a high-flow main channel, the method can monitor both aggregation behavior and flow-response changes without reference-fluid infusion. Therefore, it reduces hemodilution-related artifacts and provides a practical sensing strategy for detecting hemorheological alterations caused by hematocrit- or medium-dependent changes in continuously delivered blood.

## 2. Materials and Methods

### 2.1. Microfluidic Chip Fabrication and Experimental Setup

A novel microfluidic platform was suggested for probing the viscosity-sensitive time constant and RBC aggregation index in continuous pulsatile blood flow.

**Figure 1:**
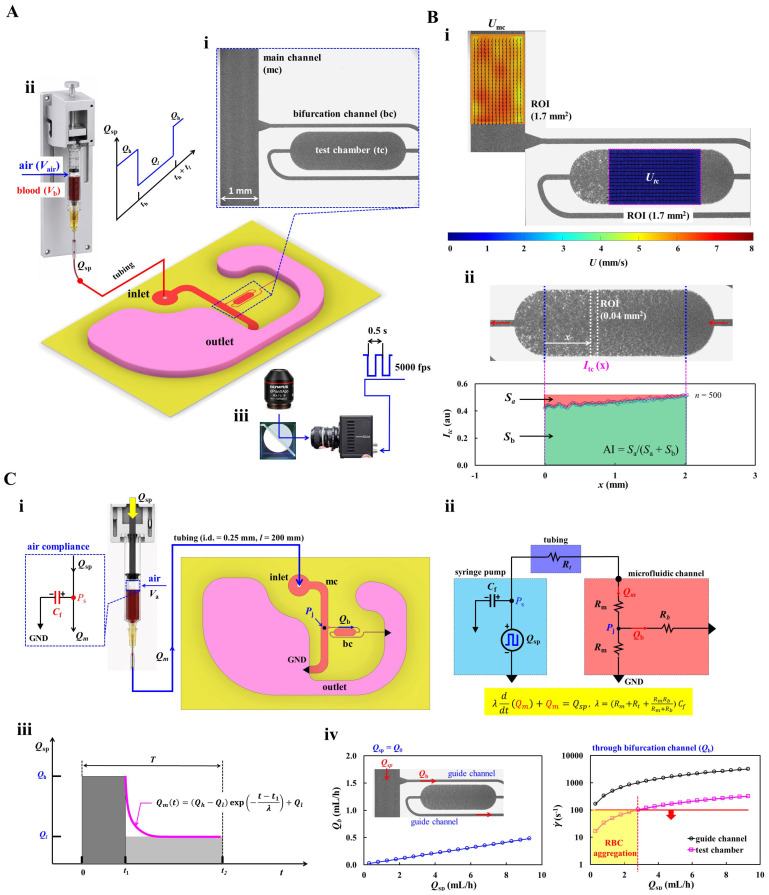
A novel microfluidic platform for probing viscosity-sensitive time constant and RBC aggregation index in continuous pulsatile blood flow. (**A**) Experimental setup, including a microfluidic chip, single syringe pump, and microscopic imaging system. (**i**) The microfluidic chip consisted of an inlet, a uniform main channel (mc) (width = 1 mm, length = 14.9 mm), a bifurcation channel, and a large outlet, with all channels terminated at the outlet. (**ii**) Single syringe pump for delivering blood into the microfluidic chip. To induce air compliance, an air cavity (*V*_air_) was maintained above blood volume (*V*_b_) inside the syringe. Blood flow rate (Q_sp_) was regulated as a function of time. (**iii**) A microscopic imaging system comprising a microscope (4× objective, NA = 0.1), a high-speed camera (5000 frames per second [fps]), and a function generator (trigger period = 0.5 s). (**B**) Quantification of blood velocity (main channel: *U_mc_*, test chamber: *U*_tc_), and image intensity (*I*_tc_) inside the test chamber. (**i**) Variations in *U_mc_* and *U_tc_* obtained from redefined regions of interest (ROI) (1.7 mm^2^) in each channel. (**ii**) Spatial distributions of *I_tc_* (x) evaluated within a smaller ROI (0.04 mm^2^) in the test chamber. Red arrows (‘←’) indicated the direction of blood flow from right to left. (**C**) Mathematical model of the proposed method and shear rate estimation in bifurcation channel. (**i**) Air compliance (*C*_f_) model of air cavity secured inside the syringe. The pressures at the junction and outlet were denoted as *P*_j_ and GND (‘▼’), respectively. (**ii**) A discrete fluidic circuit model developed for the proposed microfluidic system. (**iii**) Transient behavior of *Q*_m_ under pulsatile flow of *Q*_sp_. (**iv**) Variation in shear rate in bifurcation channels with respect to constant value of *Q*_sp_. The left panel showed variations in *Q*_b_ with respect to *Q*_sp_. The right-side panel depicted the corresponding shear rate profiles in the test chamber and guide channel, which were then rinsed with 1× PBS to remove residual BSA before blood infusion. The syringe was mounted on a syringe pump (neMESYS, Cetoni Gmbh, Korbussen, Germany). The pump was programmed to generate a pulsatile blood flow rate profile. The flow rate (*Q*_sp_) was set to *Q*_sp_ = *Q*_h_ for the high-flow interval (0 < *t* < *t*_h_) and *Q*_sp_ = *Q*_l_ mL/h for the low-flow interval (*t*_h_ < *t* < *T*), with one cycle period defined as *T* = *t*_h_ + *t*_l_.

As shown in Figure 1A, the experimental setup consisted of a microfluidic chip, a single syringe pump, and a microscopic imaging system. As shown in Figure 1A(i), the microfluidic chip comprised a single inlet, a rectangular main channel (mc) (width = 1 mm, length = 14.9 mm), a bifurcation channel, and a large outlet. The main and bifurcation channels were connected to the outlet. The bifurcation channel contained a large test chamber (tc) (width = 1 mm, length = 2 mm) positioned between two narrow guide channels (width = 0.1 mm, length = 6.89 mm). The channel depth was fixed at *h* = 50 μm throughout the device.

The microfluidic device was fabricated by PDMS replica molding from a silicon master, which was patterned using conventional photolithography and etched by deep reactive ion etching, following standard soft-lithography and MEMS micromachining procedures [36,37]. For PDMS replica molding, Sylgard 184 elastomer base and curing agent (Dow Corning, Midland, MI, USA) were mixed at a 10:1 weight ratio. The uncured PDMS mixture was degassed in a vacuum chamber for 1 h and subsequently cured at 65 °C for 2 h. After curing, the PDMS replica was gently peeled off from the silicon master, trimmed to the required dimensions, and punched to form an inlet port with an outer diameter of 2 mm. A large outlet port was also created using a punch with an outer diameter of 3 mm. Finally, the PDMS layer was irreversibly sealed onto a glass substrate after oxygen plasma activation using a plasma treatment system (CUTE-MPR, Femto Science Co., Ltd., Hwaseong-si, Republic of Korea). The assembled chip was further heated at 120 °C for 10 min to enhance the bonding strength between the PDMS and glass surfaces.

As shown in Figure 1A(ii), a single syringe equipped with a 20-gauge needle was loaded with blood (*V*_b_ = 0.5 mL). To introduce air compliance into the blood-delivery system, an air cavity (*V*_air_) was maintained above the blood column inside the syringe [38]. A trapped gas volume can function as a hydraulic capacitor that absorbs flow fluctuations and modifies the transient response of syringe-pump-driven microfluidic systems [39,40,41,42,43]. The needle tip was connected to one end of polyethylene tubing (inner diameter = 0.25 mm, length = 200 mm), while the other end of the tubing was inserted into the inlet port of the microfluidic device. To minimize nonspecific protein adsorption, the microchannels were pretreated with 0.2% bovine serum albumin (BSA) for 10 min. The channels were then rinsed with 1× PBS to remove residual BSA before blood infusion. The syringe was mounted on a syringe pump (neMESYS, Cetoni Gmbh, Korbussen, Germany). The pump was programmed to generate a pulsatile blood flow rate profile. The flow rate (*Q*_sp_) was set to *Q*_sp_ = *Q*_h_ for the high-flow interval (0 < *t* < *t*_h_) and *Q*_sp_ = *Q*_l_ mL/h for the low-flow interval (*t*_h_ < *t* < *T*), with one cycle period defined as *T* = *t*_h_ + *t*_l_.

The microfluidic chip was mounted on an inverted microscope (IX81, Olympus, Tokyo, Japan) equipped with a 4× objective lens (NA = 0.1). Microscopic images of blood flow were acquired at 5000 frames per second (fps) using a high-speed camera (FASTCAM MINI, Photron, Tokyo, Japan). Image acquisition was triggered at 0.5 s intervals using a function generator. All experiments were performed at room temperature (25 °C).

### 2.2. Quantification of Blood Velocity and Microscopic Image Intensity

Blood velocity was measured separately in the main channel and test chamber. The velocity response in the main channel was used to determine the viscosity-sensitive time constant, while the velocity in the test chamber was used to estimate the low-shear-rate condition for RBC aggregation. RBC aggregation was then quantified from the spatial distribution of image intensity along the test chamber.

First, as shown in Figure 1B(i), to obtain the average velocity in each channel, the region-of-interest (ROI) (1.7 mm^2^) was defined in the upper portion of the main channel and in the straight section of the test chamber, respectively. Velocity fields were obtained from time-lapse image sequences using PIVlab (version 3.12) [44] with an interrogation window of 129 × 129 µm^2^ and 50% overlap. The calculated velocity vectors were subsequently filtered using local median and standard deviation filters. For the optical measurement system used in this study, the depth of correlation (DOC) was estimated to exceed 300 µm, which was substantially greater than the channel depth of 50 µm [45]. According to the micro-PIV depth-of-correlation theory [46,47], all RBCs located through the illuminated depth direction could contribute to the recorded correlation signals. Therefore, the measured velocity vectors were regarded as depth-averaged velocities across the channel depth. Within each channel, the velocity vectors were spatially averaged over the selected ROI. The resulting time-dependent mean velocities in the main channel and test chamber were defined as *U*_mc_ and *U*_tc_, respectively.

Second, the initial background image was subtracted from each acquired image, and the resulting images were analyzed using MATLAB (version 2025b, MathWorks, Natick, MA, USA). As shown in the upper panel of Figure 1B(ii), a small ROI (0.04 mm^2^) was selected within the straight region of the test chamber and translated along the x-direction from the left boundary to the right boundary. The mean grayscale intensity at each position was defined as (*I*_tc_ [x]). The arrow indicated blood flow direction left to right. As blood moved from the narrow guide channel into the wider test chamber, local shear rate decreased substantially. RBC aggregation was continuously induced under low-shear conditions. As shown in the lower panel of Figure 1B(ii), the *I*_tc_ (x) gradually increased along the 2 mm chamber length, reflecting the progression of RBC aggregation. The spatial intensity distribution was then used to determine two parameters (S_a_, S_b_), following the conventional aggregation-index definition [18,21,48], and the RBC aggregation index was calculated as AI = *S*_a_/(*S*_a_ + *S*_b_).

### 2.3. A Lumped Parameter Model for Estimating Time Constant and Shear Rate

As shown in Figure 1C, a simplified mathematical model was established to estimate the viscosity-dependent time constant and shear rate in the test chamber, under pulsatile blood flow conditions.

As shown in Figure 1C(i), an air cavity was maintained above the blood volume inside the syringe. *Q*_sp_ and *Q*_m_ denoted the flow rate of the syringe pump and the flow rate through tubing, respectively. From the mass conservation law [49], the temporal change in the air cavity could be expressed as,

(1) $$\frac{d}{dt}(V_{air})={-Q}_{sp}+Q_{m}$$

Assuming that the air cavity follows the ideal-gas law [50], the relationship between the initial and instantaneous air cavity volume was given by *P*_0_ *V*_0_ = P_s_ *V*_air_, where *P*_0_ was the atmosphere pressure, *V*_0_ was initial air cavity, and *P*_s_ was air pressure inside the syringe. Thus, the instantaneous air cavity was written as *V*_air_ = *P*_0_*V*_0_/*P*_s_. Differentiating *V*_air_ with respect to time gave Equation (2) [43],

(2) $$\begin{array}{ll} \frac{d}{dt}(V_{air}) & =\frac{d}{dP_{s}}(\frac{P_{0}V_{0}}{P_{s}})\frac{dP_{s}}{dt},\\ & \;\;\;=-C_{f}\frac{dP_{s}}{dt} \end{array}$$

In Equation (2), the compliance coefficient (*C*_f_) was defined as

(3) $$C_{f}=\frac{P_{0}V_{0}}{{P_{s}}^{2}}$$

Equation (1) was then simplified in Equation (4) as

(4) $$Q_{sp}=Q_{m}+C_{f}\frac{dP_{s}}{dt}$$

Equation (4) indicated that the imposed syringe-pump flow rate was divided into the flow delivered through the tubing and the flow associated with compression or expansion of the air cavity [51]. Based on this relationship, the air cavity was represented as a compliance element in the equivalent fluidic circuit shown in the left panel of Figure 1C(i). In the circuit, GND (▼) denoted zero value of gauge pressure. *P*_j_ represented pressure at the junction point where the bifurcation channel branched from the main channel. *Q*_b_ denoted the flow rate through the bifurcation channel. Based on the Hagen–Poiseuille law (pressure drop [Δ*P*] = flow rate [*Q*] × fluidic resistance [*R*]) [49], frictional loss in the channel or tubing was represented as a fluidic resistance element (*R*). As shown in Figure 1C(ii), a lumped fluidic circuit model was established for the proposed microfluidic system. The model consisted of a syringe pump, a tubing, and a microfluidic channel. The syringe pump was represented by a flow-rate source coupled with a compliance element (*C*_f_), whereas the tubing was modeled as a hydraulic resistance (*R*_t_). The microfluidic channel was described using the hydraulic resistances of the main channel and bifurcation channel, which were denoted as *R*_m_ and *R*_b_, respectively. Herein, *R*_b_ represented the equivalent hydraulic resistance of the two guide channels and the large test chamber connected in series. By applying mass conservation at junction (*j*), the transient flow response through the main channel could be expressed as

(5) $$\lambda_{1}\frac{d}{dt}(Q_{m})+Q_{m}=Q_{sp},$$

where *Q*_sp_ was the flow rate imposed by the syringe pump and *Q*_m_ was the flow rate through the main channel. The corresponding time constant was given by

(6) $$\lambda_{1}=C_{f}(R_{m}+R_{t}+\frac{R_{m}R_{b}}{R_{m}+R_{b}}).$$

Since the equivalent hydraulic resistance was directly dependent on the apparent viscosity of blood, an increase in blood viscosity led to an increase in the flow-response time constant under the same air compliance. Therefore, the flow-response time constant could provide a physically grounded and experimentally accessible index for assessing viscosity-related hemorheological variations in the proposed microfluidic system.

As shown in Figure 1C(iii), the time-dependent syringe pump flow rate (*Q*_sp_) was defined as *Q*_sp_ = *Q*_h_ for 0 < *t* < *t*_1_ and *Q*_sp_ = *Q*_l_ for *t*_1_ < *t* < *T*. To minimize the influence of RBC aggregation on the blood viscosity, the time constant was determined under sufficiently high shear rate conditions ($\dot{\gamma }$ > 10^3^ s^−1^). Therefore, within one period (*T*), the analytical solution for *Q*_m_ was derived for the transient interval, when the *Q*_sp_ was changed from *Q*_h_ to *Q*_l_. Under this transient flow-rate condition, solving Equation (5) provided *Q*_m_ as

(7) $$Q_{m}(t)=(Q_{h}-Q_{l})exp(-\frac{t-t_{1}}{\lambda_{1}})+Q_{l}.$$

In Equation (7), $Q_{m}(t)$ in the main channel could be expressed as $Q_{m}(t)=U_{mc}(t)A_{mc}$, where *U*_mc_ was the time-dependent mean velocity and *A*_mc_ was the cross-sectional area of the main channel. The time constant (*λ*_1_) was obtained by regression analysis of the time-lapse *U*_mc_ (*t*) data during the flow-rate transition from *Q*_h_ to *Q*_l_.

To estimate the shear rate in the test chamber, the syringe flow rate was assumed as a constant value of *Q*_sp_ = *Q*_0_. Based on mass conservation at the junction point (‘*j*’), pressure at the junction (*P*_j_) was derived as

(8) $$P_{j}=\frac{R_{m}R_{b}}{R_{m}+R_{b}}Q_{sp}.$$

Dividing *P*_j_ by *R*_b_ gave the expression for *Q*_b_ as

(9) $$Q_{b}=\frac{Q_{sp}}{1+\frac{R_{b}}{R_{m}}}.$$

With regard to the microfluidic channel proposed in this study, the fluidic resistance ratio (*R*_b_/*R*_m_) was calculated as *R*_b_/*R*_m_ = 18.76. As shown in the left panel of Figure 1C(iv), variations in flow rate in the bifurcation (*Q*_b_) were estimated by increasing *Q*_sp_ ranging from 0.5 mL/h to 9.5 mL/h. As expected, the *Q*_b_ was linearly proportional to *Q*_sp_. In the inset of Figure 1C(iv), the arrow (‘←’) denoted blood flow direction in the bifurcation channel. The right-side panel depicted the corresponding shear rate profiles in the test chamber and guide channel, respectively. Considering that RBC aggregation was mainly promoted under low shear rate conditions ($\dot{\gamma }<100\;s^{-1}$) [5,25,52,53,54], RBC aggregation could be observed in the yellow region (i.e., test chamber), which corresponded to *Q*_sp_ < 3 mL/h. In contrast, no appreciable RBC aggregation was detected within the guide channel.

### 2.4. Test Blood Preparation

A packed RBC unit was provided by the Gwangju–Chonnam Blood Bank (Gwangju, Republic of Korea) and maintained at a refrigerated temperature before experiments. For sample preparation, normal RBCs were collected using an established washing protocol [55,56]. After centrifugation, the supernatant containing the storage medium and washing buffer was carefully aspirated, and the buffy coat layer was subsequently removed to minimize leukocyte and platelet contamination. The RBC pellet was then resuspended in 1× PBS, and this washing procedure was repeated twice to minimize residual storage solution in the RBC suspension.

To systematically characterize the flow-response time constant and RBC aggregation, two different sets of test blood were prepared by varying hematocrit and blood medium. First, the effect of hematocrit (*ϕ*_vol_) was examined using normal RBCs suspended in 1% dextran solution, where hematocrit was set from 20% to 60%. The dextran solution was prepared by dissolving dextran powder (Leuconostoc spp., MW 450–650 kDa, Sigma-Aldrich, St. Louis, MO, USA) into 1× PBS. Second, the medium-dependent response was evaluated by varying the dextran concentration from 1% to 4% at a fixed hematocrit of *ϕ*_vol_ = 50%.

### 2.5. Statistical Analysis

Statistical analyses were performed using MINITAB software (version 22.4, Minitab Inc., State College, PA, USA) and Microsoft Excel 365 (Microsoft, Redmond, WA, USA). Assuming normal distribution, all measured values were presented as mean ($\overset{–}{x}$) ± standard deviation (σ). Sample size was denoted as *n*. 95% confidence interval (CI) was estimated calculated using $\overset{–}{x}$ − 1.96 σ/$\sqrt{n}$ and $\overset{–}{x}$ + 1.96 σ/$\sqrt{n}$ as the lower and upper limits, respectively. Differences among groups were evaluated using one-way ANOVA. Statistical significance was defined as *p*-value < 0.05. Linear regression analysis was performed to assess the correlation between paired variables.

## 3. Results and Discussion

### 3.1. Demonstration of the Proposed Method

To demonstrate the proposed method, the viscosity-sensitive time constant and RBC aggregation index (AI) were obtained for the control blood and test blood. Herein, the hematocrit of both bloods (test blood, control blood) was adjusted to *ϕ*_vol_ = 0.5 by adding normal RBCs into dextran (*C*_dex_ = 2%) and 1× PBS, respectively. The air cavity (*V*_air_ = 0.1 mL) was maintained above blood column (V_b_ = 0.5 mL) inside the syringe. The syringe pump was set to generate a pulsatile flow profile (i.e., *Q*_h_ = 6 mL/h for 0 < *t* < 2 min, *Q*_l_ = 1 mL/h for 2 min < *t* < 6 min, and *T* = 6 min).

**Figure 2:**
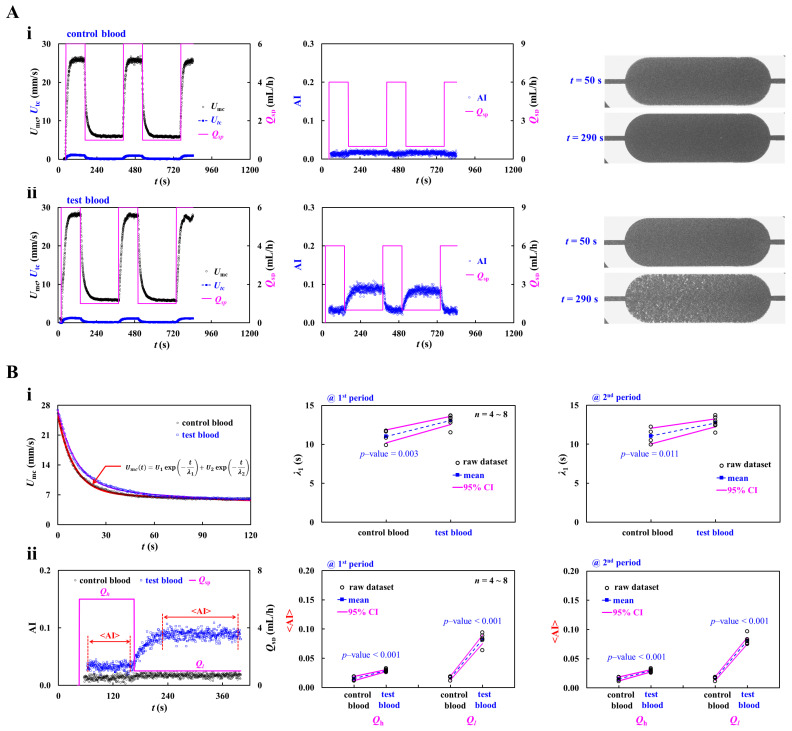
Demonstration of the proposed method for detecting control and test bloods with viscosity-sensitive time constant and RBC aggregation index. (**A**) Quantitative comparison of timelapse blood velocity and RBC aggregation index under pulsatile blood flow. (**i**) Temporal variations in blood velocity and RBC aggregation index for control blood. The left panel represented temporal variations in blood velocity (*U*_mc_, *U*_tc_) and *Q*_sp_. The middle panel depicted temporal variations in RBC aggregation index (AI) and *Q*_sp_. The right panel showed microscopic blood-flow images in the test channel captured at *t* = 50 s and 290 s. (**ii**) Temporal variations in blood velocity, and RBC aggregation index for test blood. The left panel represented timelapse *U*_mc_, *U*_tc_, and *Q*_sp_. The middle panel depicted time-dependent AI and *Q*_sp_. The right panel showed microscopic blood-flow images in test channel captured at *t* = 50 s and 290 s. (**B**) Quantification of time constant and RBC aggregation over period. (**i**) Quantification of time constant using time-lapse *U*_mc_ during two consecutive periods. The left panel showed timelapse *U*_mc_ for transient blood flow, where the syringe pump was switched to *Q*_l_ from *Q*_h_. The middle panel showed λ_1_ for two blood samples during the first period. The right panel exhibited λ_1_ for two blood samples during the second period. (**ii**) Quantification of AI for two blood samples during two consecutive periods. The left panel showed time-dependent AI for two blood samples. Herein, the mean AI (<AI>) was determined by averaging AI values within the plateau region observed at both *Q*_h_ and *Q*_l_. The middle panel depicted <AI> for two blood samples under *Q*_h_ and *Q*_l_ during the first period. The right panel exhibited <AI> for both samples under *Q*_h_ and *Q*_l_ during the second period.

As shown in Figure 2A, the time-lapse RBC aggregation index was obtained for control and test bloods. Figure 2A(i) showed temporal variations in the blood velocity and RBC aggregation index for control blood. The left panel represented temporal variations in blood velocity (*U*_mc_, *U*_tc_) and *Q*_sp_. The middle panel depicted temporal variations in the RBC aggregation index (AI) and *Q*_sp_. The right panel showed microscopic blood-flow images in the test channel captured at *t* = 50 s and 290 s. As the control blood did not induce RBC aggregation in the test chamber, the microscopic image did not exhibit substantial variations. In contrast, control blood was replaced by test blood. As shown in Figure 2A(ii), temporal variations in blood velocity and RBC aggregation index were acquired for the test blood. The left panel represented time-lapse *U*_mc_, *U*_tc_, and *Q*_sp_. When the syringe flow rate was switched from *Q* = 6 mL/h to *Q* = 1 mL/h, the test blood exhibited a longer transient response than the control blood. The middle panel depicted time-dependent AI and *Q*_sp_. The aggregation index (AI) increased substantially under the low flow condition (*Q* = 1 mL/h), whereas it remained relatively low at the high flow rate (*Q* = 6 mL/h). The right panel showed microscopic blood-flow images in the test channel captured at *t* = 50 s and 290 s. At the high flow rate (*t* = 50 s), no appreciable morphological difference was observed between the test and control bloods. However, at the low flow rate (*t* = 290 s), pronounced RBC aggregation led to a marked increase in cell-free void regions.

As shown in Figure 2B, the time constant and RBC aggregation index (AI) of the control and test bloods were quantitatively evaluated during the first and second periods. Figure 2B(i) exhibited the time constant obtained from the time-lapse *U*_mc_ data over two consecutive periods. The left panel showed the temporal variation in *U*_mc_ for both bloods during the stepwise decrease in the syringe-pump flow rate from *Q*_h_ to *Q*_l_. The results indicated that the decrease in *U*_mc_ was slower for the test blood than for the control blood. Compared with the control blood, the test blood exhibited a slower decrease in *U*_mc_, indicating a longer transient response in the test blood. To accurately describe the transient behavior of *U*_mc_, the time-lapse velocity data were fitted using a two-exponential model, $U_{mc}(t)=U_{1}exp(-\frac{t}{\lambda_{1}})+U_{2}exp(-\frac{t}{\lambda_{2}})$. In the bi-exponential model, λ_1_ denoted the fast time constant corresponding to the primary transient response immediately after flow-rate modulation. By contrast, λ_2_ denoted a slower secondary relaxation component, which might be influenced by residual compliances resulting from flexible tubing, and PDMS microfluidic channels. Non-linear curve-fitting was performed using a curve-fitting toolbox in Matlab. The resulting fitting equations were expressed as $U_{mc}(t)=18.41\;exp(-\frac{t}{9.88\;})+7.41\;exp(-\frac{t}{476.19\;})$ for control blood and $U_{mc}(t)=18.44\;exp(-\frac{t}{11.49})+8.45\;exp(-\frac{t}{312.5})$ for test blood, respectively. Because $\lambda_{1}$ was much smaller than $\lambda_{2}$, $\lambda_{1}$ was selected as the representative time constant characterizing the dominant rapid transient response. The higher $\lambda_{1}$ value of the test blood indicated that its transient flow response was slower than that of the control blood. The middle panel showed the λ_1_ values obtained for the two bloods during the first period. Each blood was tested with *n* = 4~8 replicates. The mean value and 95% confidence interval (CI) were superimposed on the raw data points. The λ_1_ value of the test blood was substantially higher than that of the control blood. One-way ANOVA confirmed a statistically significant difference between two groups (*p*-value = 0.003). The right panel presented the λ_1_ values obtained for the two bloods during the second period. One-way ANOVA revealed a statistically significant difference between the control and test bloods (*p*-value = 0.011). Similarly, as shown in Figure 2B(ii), the RBC aggregation index (AI) of the control and test bloods was quantitatively evaluated during two consecutive periods. The left panel showed time-dependent AI and Q_sp_ for both bloods during the first period. Herein, the mean AI (<AI>) was calculated by averaging the AI values within the plateau regions observed at both *Q*_h_ and *Q*_l_. The middle panel presented the mean AI values (<AI>) of the control and test bloods at *Q*_h_ and *Q*_l_ during the first period. One-way ANOVA confirmed statistically significant differences between the two bloods at both flow rates (*p*-value < 0.001). Notably, the difference in <AI> between two bloods was more pronounced at a low flow rate (*Q*_l_). The right panel exhibited the mean AI (<AI>) values for both samples at *Q*_h_ and *Q*_l_ during the second period. One-way ANOVA indicated significant differences between two bloods at both flow rates (*p*-value < 0.001).

The preliminary results showed that the proposed single syringe pump microfluidic method could simultaneously assess viscosity-related flow dynamics and RBC aggregation under periodically modulated flow conditions. Compared with the control blood, the test blood exhibited a significantly higher time constant (*λ*_1_) and aggregation index (AI), indicating increased flow resistance and enhanced RBC aggregation, particularly under the low-flow-rate condition (*Q*_l_).

### 3.2. Correlation Between Blood Viscosity and Time Constant

In this subsection, to probe the linear relationship between time constant and blood viscosity, the blood flow rate was adjusted to ensure a sufficiently high shear condition of $\dot{\gamma }$ > 10^3^ s^−1^. Under these high shear regimes, blood viscosity could be reasonably treated as constant. According to Equation (6), the time constant (λ_1_) was linearly proportional to blood viscosity. That is, the hydraulic resistance of a fixed microfluidic channel was linearly proportional to fluid viscosity under laminar flow, while the transient response of a compliant fluidic system was governed by a hydraulic resistance–compliance time constant [42,57,58,59,60]. When the air volume and channel geometry were maintained at a constant, the compliance term remained nearly unchanged and the measured time constant (*λ*_1_) was expected to vary linearly with blood viscosity. The linear relationship between the time constant and blood viscosity was experimentally validated by varying the infusion flow rate, hematocrit, and dextran concentration. Herein, the air cavity inside the syringe was fixed at *V*_air_ = 0.1 mL.

**Figure 3:**
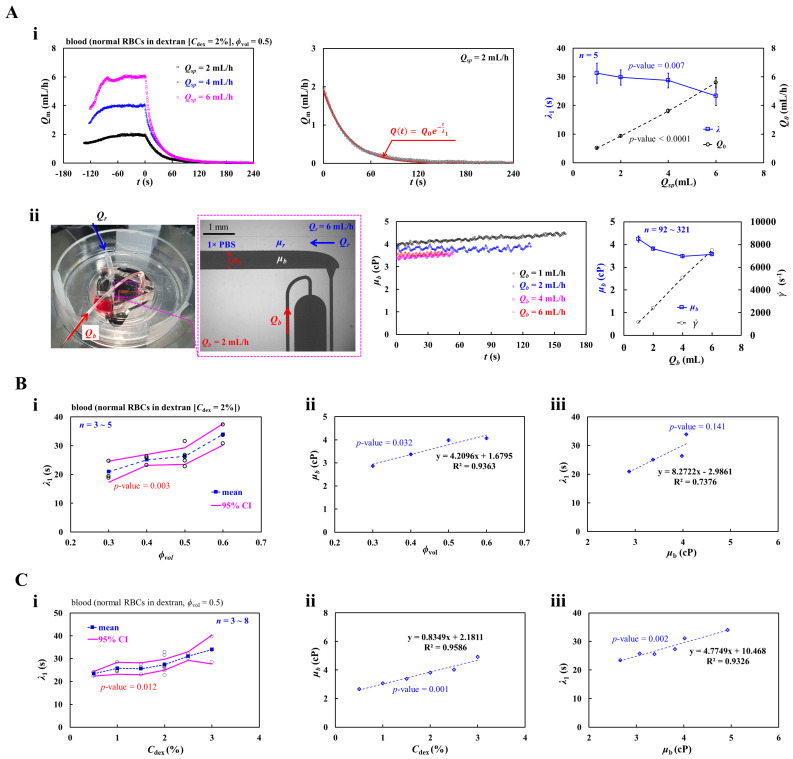
Validation of correlation between blood viscosity and the time constant. (**A**) Quantification of time constant and blood viscosity. (**i**) Determination of time constant through transient blood flow analysis in the main channel (*Q*_m_). The left panel exhibited time-dependent *Q*_m_ with respect to plateau flow rates of *Q*_sp_ = 2, 4, and 6 mL/h. The middle panel showed timelapse *Q*_m_ at *Q*_sp_ = 2 mL/h. The right panel showed variations in *λ*_1_ and *Q*_0_ with respect to plateau flow rates of *Q*_sp_. (**ii**) Blood viscosity measurement with coflowing method. The left panel showed the experimental setup and microscopic image for measuring blood viscosity (*μ*_b_). The middle panel showed time-dependent blood viscosity with respect to constant flow-rate of *Q*_b_. The right panel exhibited variation in *μ*_b_ and $\dot{\gamma }$ with respect to *Q*_b_. (**B**) Quantitative correlation between blood viscosity and time constant with respect to hematocrit (*ϕ*_vol_). (**i**) Variations in time constant (λ_1_) with respect to *ϕ*_vol_. (**ii**) Variations in blood viscosity (*μ*_b_) with respect to *ϕ*_vol_. (**iii**) Linear correlation between λ_1_ and *μ*_b_. (**C**) Quantitative correlation between blood viscosity and time constant with respect to dextran (*C*_dex_). Test blood (*ϕ*_vol_ = 0.5) was prepared by adding normal RBCs into dextran (*C*_dex_ = 0.5~3%). (**i**) Variations in time constant (λ_1_) with respect to *C*_dex_. (**ii**) Variations in blood viscosity (*μ*_b_) with respect to *C*_dex_. (**iii**) Linear correlation between λ_1_ and *μ*_b_ was obtained by substituting blood-filled width (*α*_b_) into Equation (10). The middle panel showed time-dependent blood viscosity with respect to a constant flow-rate of *Q*_b_ = 1, 2, 4, and 6 mL/h. Blood viscosity remained constant over time and decreased at a higher flow rate of *Q*_b_. The right panel exhibited variation in *μ*_b_ and $\dot{\gamma }$ with respect to *Q*_b_. Herein, the shear rate formula of a rectangular microfluidic channel was given as $\dot{\gamma }\;$ = $\frac{6\;Q_{b}}{w\;h^{2}}$. From the results, the *μ*_b_ was decreased gradually for up to *Q*_b_ = 4 mL/h, where the $\dot{\gamma }$ was calculated as about 5089 s^−1^. Blood viscosity remained constant at *Q*_b_ = 4~6 mL/h.

As shown in Figure 3A, time constant and blood viscosity were acquired as a function of flow rate (*Q*_sp_) (*Q*_sp_ = 1~6 mL/h). Herein, test blood (*ϕ*_vol_ = 0.5) was prepared by adding normal RBCs into dextran (*C*_dex_ = 2%). Syringe pump was abruptly stopped from the plateau value of *Q*_sp_ = 1, 2, 4, and 6 mL/h. As shown in Figure 3A(i), the time constant of test blood was obtained quantitatively from time-lapse blood flow rate in the main channel (*Q*_m_). The left panel exhibited time-dependent *Q*_m_ with respect to the plateau value of *Q*_sp_ = 2, 4, and 6 mL/h. As shown in the middle panel, time-lapse *Q*_m_ was redrawn from the onset of transient blood flow. Herein, the plateau value of *Q*_sp_ was set to *Q*_sp_ = 2 mL/h. Based on one exponential model, $Q_{m}(t)\;=\;Q_{0}exp\;(-\frac{t}{\lambda_{1}}$), the time-lapse *Q*_m_ was best described by $Q_{m}(t)\;=\;1.92\;exp\;(-\frac{t}{27.62}$). Amplitude and time constant were obtained as *Q*_0_ = 1.92 mL/h and *λ*_1_ = 27.62 s, respectively. The right panel showed variations in *λ*_1_ and *Q*_0_ with respect to the plateau value of *Q*_sp_. The experiment was repeated five times (*n* = 5) at each flow rate. The *Q*_0_ increased linearly with respect to the plateau flow rate of *Q*_sp_ (*p*-value < 0.001), while λ_1_ decreased gradually for up to *Q*_sp_ = 4 mL/h and dropped substantially at the plateau flow rate of *Q*_sp_ = 6 mL/h (*p*-value < 0.001). As shown in Figure 3A(ii), a coflowing stream method was adopted to measure blood viscosity at a plateau value of *Q*_sp_ = 1~6 mL/h. The left panel showed experimental setup and microscopic image for measuring blood viscosity (*μ*_b_). According to the previous study [29], the viscosity formula of test fluid was given as

(10) $$\mu_{b}=\mu_{r}\left(\frac{\alpha_{b}}{1-\alpha_{b}}\right)\left(\frac{Q_{r}}{Q_{b}}\right)C_{f}(\alpha_{b}),$$

where viscosity of reference fluid (1× PBS) was denoted as $\mu_{r}=1\;cP.$ The formula for the correction factor, *C*_f_ (*α*_b_), was obtained experimentally and derived as a polynomial expression,

(11) $$C_{f}=12.038\;{\alpha_{b}}^{4}+26.171\;{\alpha_{b}}^{3}-20.77{\alpha_{b}}^{2}+7.156{\;\alpha }_{b}+0.014.$$

Herein, test blood was set to *Q*_b_ = 2 mL/h. To relocate the interface near the channel center, the flow rate of the reference fluid (1× PBS) was adjusted to *Q*_r_ = 6 mL/h. Then, blood viscosity was obtained by substituting blood-filled width (*α*_b_) into the Equation (10). The middle panel showed time-dependent blood viscosity with respect to constant flow-rate of *Q*_b_ = 1, 2, 4, and 6 mL/h. Blood viscosity remained constant over time and decreased at higher flow rate of *Q*_b_. The right panel exhibited variation of *μ*_b_ and $\dot{\gamma }$ with respect to *Q*_b_. Herein, shear rate formula of a rectangular microfluidic channel was given as $\dot{\gamma }\;$ = $\frac{6\;Q_{b}}{w\;h^{2}}$. From the results, the *μ*_b_ was decreased gradually for up to *Q*_b_ = 4 mL/h, where the $\dot{\gamma }$ was calculated as about 5089 s^−1^. Blood viscosity was remained constant at *Q*_b_ = 4~6 mL/h.

As shown in Figure 3B, the correlation between blood viscosity and time constant was validated by varying hematocrit (*ϕ*_vol_). Herein, hematocrit of test blood was adjusted to *ϕ*_vol_ = 0.3~0.6 by adding normal RBCs into dextran (*C*_dex_ = 2%). To probe time constant consistently, blood flow rate was abruptly stopped from plateau value of *Q*_b_ = 2 mL/h. Under transient blood flow, as shown in Figure 3B(i), time constant (λ_1_) was obtained as a function of *ϕ*_vol_. Multiple experiments for each hematocrit were carried out (*n* = 3~5). The time constant (λ_1_) increased remarkably with respect to hematocrit (*p*-value = 0.003). In addition, as shown in Figure 3B(ii), blood viscosity (*μ*_b_) was obtained with respect to *ϕ*_vol_. To examine the relationship between two parameters, time constant (*λ*_1_) and blood viscosity (*μ*_b_) were plotted on the y-axis and x-axis, respectively. As shown in Figure 3B(iii), *λ*_1_ tended to increase with increasing *μ*_b_. Linear regression analysis yielded the following relationship: *λ*_1_ = 8.2722 *μ*_b_ − 2.9861 (R^2^ = 0.7376, *p*-value = 0.141). Although the correlation did not reach statistical significance, the result suggested a positive association between *λ*_1_ and *μ*_b_, supporting the potential use of the time constant as a viscosity-related parameter.

As shown in Figure 3C, correlation between blood viscosity and time constant was evaluated as a function of dextran concentration (*C*_dex_). For this analysis, test blood (*ϕ*_vol_ = 0.5) was prepared by suspending normal RBCs into dextran solution (*C*_dex_ = 0.5~3%). Under transient blood flow conditions, as shown in Figure 3C(i), time constant (λ_1_) was determined as a function of dextran concentration (*C*_dex_). The time constant (*λ*_1_) increased markedly with increasing dextran concentration, showing a statistically significant dependency on the dextran concentration (*p*-value = 0.012). Under steady blood flow conditions, as represented in Figure 3C(ii), blood viscosity (*μ*_b_) was measured with respect to *C*_dex_. The linear regression formula was obtained as *μ*_b_ = 0.8349 *C*_dex_ + 2.1811 (R^2^ = 0.9586, *p*-value = 0.001), indicating that the *μ*_b_ increased significantly with respect to *C*_dex_. As shown in Figure 3C(iii), the *λ*_1_ was plotted against *μ*_b_. According to linear analysis, linear regression analysis yielded *λ*_1_ = 4.7749 *μ*_b_ + 10.468 (R^2^ = 0.9326, *p*-value = 0.002).

From experimental results, including variations in infusion flow rate, hematocrit, and dextran concentration, the time constant (λ_1_) increased with blood viscosity (*μ*_b_). Strong correlations between (λ_1_) and (*μ*_b_), particularly under hematocrit- and dextran-dependent changes, demonstrated that (λ_1_) could be regarded as a reliable viscosity-related parameter.

### 3.3. Validation of the Proposed RBC Aggregation Index Against the Previous Methods

**Figure 4:**
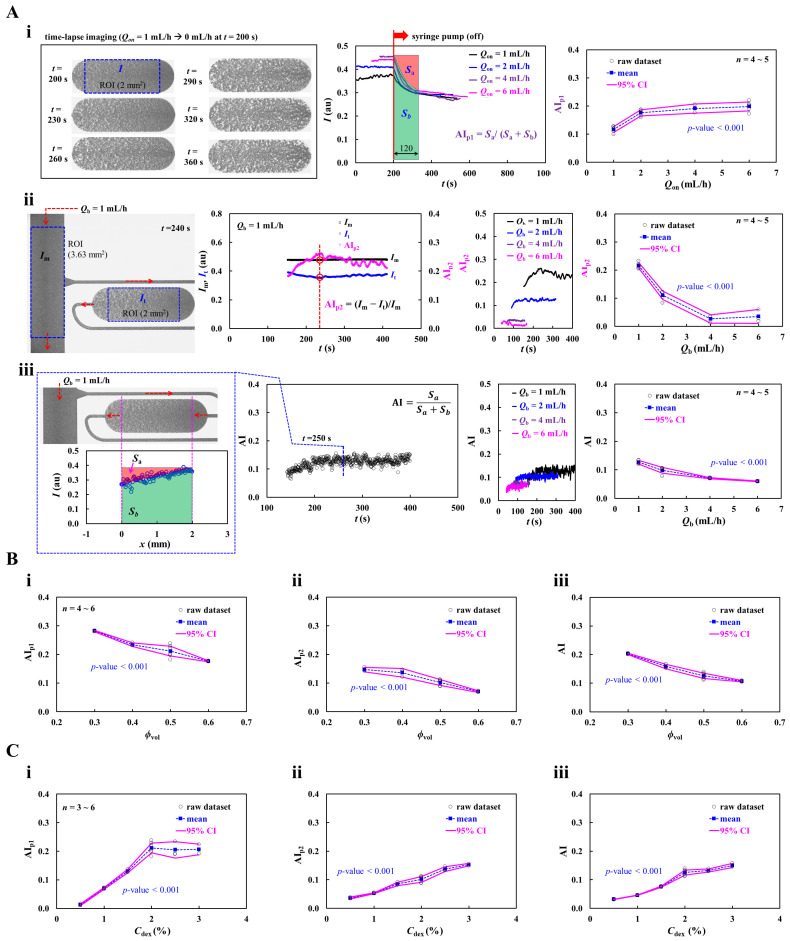
Validation of the proposed RBC aggregation index against the previous methods. (**A**) Contribution of blood flow-rate (*Q*_b_) to three quantitative methods of RBC aggregation index. (**i**) Quantification of RBC aggregation index (AI_p1_) at stasis. The left panel depicted time-resolved microscopic image captured at *t* = 200, 230, 260, 290, 320, and 360 s, where blood flow was suddenly stopped at 200 s from the plateau flow rate of *Q*_on_ = 1 mL/h. The middle panel showed time-lapse image intensity (*I*) with respect to a plateau flow rate of *Q*_on_ = 1~6 mL/h. The right panel showed variations in AI_p1_ with respect to *Q*_on_. (**ii**) Quantification of RBC aggregation index (AI_p2_) under continuous blood flow. The left panel showed microscopic image captured at *t* = 240 s, where blood flow rate was set to *Q*_b_ = 1 mL/h. The red arrow (→) denoted blood flow direction. The second panel exhibited time-lapse *I*_m_, *I*_t_, and AI_p2_ at *Q*_b_ = 1 mL/h. The third panel showed time-lapse AI_p2_ with respect to *Q*_b_ = 1~6 mL/h. The last panel depicted variations in AI_p2_ with respect to *Q*_b_. (**iii**) Quantification of RBC aggregation index (AI) obtained by the proposed method. The first panel showed blood image intensity (*I*) across the test chamber. The second panel showed time-lapse AI at *Q*_b_ = 1 mL/h. The third panel depicted time-lapse AI with respect to *Q*_b_ = 1, 2, 4, and 6 mL/h. The last panel showed variations in AI with respect to *Q*_b_. (**B**) Quantitative comparison of three RBC aggregation indices (i.e., AI_p1_, AI_p2_, and AI) with respect to hematocrit (*ϕ*_vol_). (**i**) Variation in AI_p1_ with respect to *ϕ*_vol_. (**ii**) Variation in AI_p2_ with respect to *ϕ*_vol_. (**iii**) Variation in AI with respect to *ϕ*_vol_. (**C**) Quantitative comparison of three RBC aggregation indices (i.e., AI_p1_, AI_p2_, and AI) with respect to dextran concentration (C_dex_). (**i**) Variation in AI_p1_ with respect to *C*_dex_. (**ii**) Variation in AI_p2_ with respect to *C*_dex_. (**iii**) Variation in AI with respect to *C*_dex_.

According to the previous studies, microscopic blood imaging can be effectively used to quantify the RBC aggregation index, as demonstrated through comparative studies with conventional approaches such as erythrocyte sedimentation measurement [61,62] and ultrasonic signal analysis [63]. Furthermore, the RBC aggregation index obtained under continuous blood flow has shown results comparable to those measured under periodic on–off blood flow conditions [13]. To validate the RBC aggregation index (AI) proposed in this study, its performance was compared with those of the previously reported methods [17,21,29]. As shown in Figure 4A, the AI values obtained from the three methods were quantitatively evaluated under different blood flow conditions. Test blood with a volume fraction of *ϕ*_vol_ = 0.5 was prepared by suspending normal RBCs in dextran solution at *C*_dex_ = 2%. To examine the influence of blood flow rate on AI, the plateau blood flow rate (*Q*_b_) was systematically varied from 1 to 6 mL/h.

As in the first method, RBC aggregation index (AI_p1_) was obtained by analyzing temporal variation in microscopic image intensity at stasis [15,17,21]. As shown in Figure 4A(i), the left panel presented time-resolved microscopic images acquired at *t* = 200, 230, 260, 290, 320, and 360 s. Blood flow was suddenly stopped at 200 s after reaching a plateau flow rate of *Q*_on_ = 1 mL/h. After blood flow was stopped, RBCs gradually formed aggregates over time, resulting in a pronounced reduction in image intensity. The middle panel showed time-lapse image intensity (*I*), measured at plateau flow rates of *Q*_on_ = 1, 2, 4, and 6 mL/h. According to the conventional definition of RBC aggregation index (AI_p1_), the two characteristic parameters (*S*_a_, *S*_b_) were calculated from temporal intensity profile during the first 120 s after the onset of stasis. The conventional RBC aggregation index was then computed as AI_p1_ = *S*_a_/(*S*_a_ + *S*_b_). The right panel summarized variations in AI_p1_ as a function of *Q*_on_. A substantial rise in AIp1 was observed as Q_on_ increased from 1 to 2 mL/h, and this change was found to be statistically significant (*p*-value < 0.001). At higher flow rates (*Q*_on_ > 2 mL/h), the AI_p1_ showed only a gradual increase, and no statistically significant difference was observed among the specific flow rate conditions.

More recently, our group suggested another RBC aggregation index (AI_p2_) which could be measured under continuous blood flow [13,29]. Unlike the conventional method, this approach did not require repeated stopping of blood flow. Instead, blood was continuously supplied into a microfluidic device at a constant flow rate using a syringe pump. The microfluidic device comprised a main channel and a test channel. Blood flowing through the main channel was exposed to relatively high shear conditions, under which RBC aggregates were largely dispersed. In contrast, blood entering the test channel experienced a substantially lower shear rate, promoting continuous RBC aggregation. The RBC aggregation index (AI_p2_) was then calculated based on the difference in image intensity between the two channels. The left panel showed a microscopic image captured at *t* = 240 s, when the blood flow rate was maintained at *Q*_b_ = 1 mL/h. The red arrow (→) indicated the direction of blood flow in the channels. To quantify the image intensity in the main and test channels (i.e., *I*_m_: main channel, and *I*_t_: test channel), the ROI size in each channel was set to 3.63 mm^2^ and 2 mm^2^, respectively. The AI_p2_ was then determined as AI_p2_ = (*I*_m_ − *I*_t_)/*I*_m_.

The second panel showed time-dependent *I*_m_, *I*_t_, and AI_p2_ at *Q*_b_ = 1 mL/h. From the results, the *I*_m_ remained nearly constant throughout the measurement period. In contrast, the *I*_t_ decreased gradually during the initial time, and increased slowly after reaching its minimum value. The AI_p2_ increased progressively from the initial time, reached a maximum value, and decreased gradually over time. The third panel presented time-dependent variations in AI_p2_ at different flow rates of *Q*_b_ = 1, 2, 4, and 6 mL/h. The results clearly indicated that the Q_b_ had a strong influence on AI_p2_. In particular, the AI_p2_ showed a relatively high value at the low flow rate of *Q*_b_ = 1 mL/h, whereas it decreased markedly at the higher flow rate of *Q*_b_ = 6 mL/h. This trend suggested that RBC aggregation was enhanced at the lower flow rates, whereas it was suppressed at the higher flow rates. The last panel depicted variations in AI_p2_ as a function of *Q*_b_. For each flow condition, the measurement was repeated about *n* = 4~5. The results showed that the AI_p2_ decreased markedly as *Q*_b_ increased from 1 mL/h to 4 mL/h (*p*-value < 0.001). However, no substantial difference in AI_p2_ was observed between *Q*_b_ = 4 and *Q*_b_ = 6 mL/h.

As shown in Figure 4A(iii), the RBC aggregation index (AI) proposed in this study was obtained as a function of blood flow rate. The first panel showed blood image intensity (*I*) across the test chamber under a constant flow rate of Q_b_ = 1 mL/h. The inset showed a microscopic image captured at *t* = 250 s, where the red arrow (←) indicated the direction of blood flow through the main and bifurcation channels. Two parameters (*S*_a_, *S*_b_) were obtained from the spatial variation in *I* and were subsequently used to calculate the RBC aggregation index as AI = S_a_/ (*S*_a_ + *S*_b_). The second panel presented time-dependent variation in AI at *Q*_b_ = 1 mL/h. When compared with AI_p2_, the proposed AI remained relatively stable during the measurement period of 250 s. The AI value was expressed as mean ± standard deviation. At *Q*_b_ = 1 mL/h, AI was measured as AI = 0.123 ± 0.016 (*n* = 514). The third panel depicted time-lapse AI with respect to *Q*_b_ = 1, 2, 4, and 6 mL/h. The corresponding AI values of each flow rate were obtained as AI = 0.103 ± 0.008 (*n* = 446) at *Q*_b_ = 2 mL/h, AI = 0.073 ± 0.006 (*n* = 211) at *Q*_b_ = 4 mL/h, and AI = 0.061 ± 0.008 (*n* = 214) at *Q*_b_ = 6 mL/h. The last panel summarized variations in AI with respect to *Q*_b_. The results indicated that the constant flow rate (*Q*_b_) had a significant influence on AI over the tested flow rate range (*p*-value < 0.001). Compared with previous method (AI_p2_), the proposed AI showed lower absolute values under the same flow rate conditions. However, because its temporal variation was much smaller, the proposed AI provided more stable measurements and more consistent trends with respect to the infusion flow rate.

As the first demonstration, as shown in Figure 4B, the performance of the proposed AI was evaluated by comparing three aggregation indices (i.e., AI_p1_, AI_p2_, and AI) as a function of hematocrit (*ϕ*_vol_). The hematocrit of test blood was adjusted from *ϕ*_vol_ = 0.3 to *ϕ*_vol_ = 0.6 by suspending normal RBCs into dextran solution (*C*_dex_ = 2%). The plateau flow rate was fixed at *Q*_b_ = 2 mL/h. Figure 4B(i) presented variation in AI_p1_ with respect to *ϕ*_vol_. The results indicated that AI_p1_ decreased substantially with increasing hematocrit (*p*-value < 0.001). For each hematocrit condition, experiments were repeated about *n* = 4~6. Figure 4B(ii) exhibited variation in AI_p2_ with respect to *ϕ*_vol_. Similar to AI_p1_, the AI_p2_ decreased significantly as hematocrit increased (*p*-value < 0.001). When compared with AI_p1_, the AI_p2_ exhibited lower absolute values under the same hematocrit conditions. Nonetheless, both indices showed a consistent decreasing trend with increasing hematocrit. Figure 4B(iii) depicted the variation in the proposed AI with respect to *ϕ*_vol_. The proposed AI also showed a significant dependence on hematocrit (*p*-value < 0.001). Importantly, it showed a similar trend to the two previous indices (AI_p1_, AI_p2_), supporting the reliability of the proposed AI for evaluating hematocrit-dependent changes in RBC aggregation.

In the second demonstration, shown in Figure 4C, three RBC aggregation indices (i.e., AI_p1_, AI_p2_, and AI) were compared quantitatively as a function of dextran concentration (C_dex_). Test blood (*ϕ*_vol_ = 0.5) was prepared by suspending normal RBCs into dextran solution with different concentrations (*C*_dex_ = 0.5~3%). The plateau flow rate was fixed at *Q*_b_ = 2 mL/h. Figure 4C(i) presented variation in AI_p1_ with respect to *C*_dex_. The results indicated that the AI_p1_ increased significantly as the dextran concentration increased from 0.5% to 2% (*p*-value < 0.001). However, it remained nearly constant when C_dex_ was further increased between *C*_dex_ = 2% and *C*_dex_ = 3%. Figure 4C(ii) depicted variation in AI_p2_ as a function of *C*_dex_. Unlike AI_p1_, the AI_P2_ continued to increase significantly over the entire tested dextran concentration range from *C*_dex_ = 0.5% to *C*_dex_ = 3% (*p*-value < 0.001). Figure 4C(iii) depicted variation in proposed AI with respect to *C*_dex_. The proposed AI showed a statistically significant difference across the tested dextran concentration (*p*-value < 0.001). In particular, its increasing trend was similar to that of AIp2, indicating that the proposed AI could effectively reflect dextran-induced enhancement of RBC aggregation.

From the experimental investigation, the proposed AI provided reliable and stable quantification of RBC aggregation when compared with the previous indices (AI_p1_, AI_p2_). The proposed AI showed consistent and statistically significant trends with respect to both hematocrit and dextran concentration. In particular, it decreased with increasing hematocrit and increased with increasing dextran concentration, in agreement with the established behavior of RBC aggregation [27,29,56,64]. These results demonstrate that the proposed AI could serve as a robust and reproducible index for quantifying RBC aggregation under continuous blood flow.

### 3.4. Optimization of Infusion Pulsatile Blood Flow Profile

In this subsection, a pulsatile blood flow profile was optimized to enable effective measurement of time constant and RBC aggregation index. Herein, air cavity inside the syringe was adjusted to *V*_air_ = 0.1 mL. Test blood (*ϕ*_vol_ = 0.5) was prepared by suspending normal RBCs in dextran solution (*C*_dex_ = 2%). The minimum infusion flow rate was fixed at *Q*_l_ = 1 mL/h. Three design variables (i.e., *Q*_h_: maximum flow rate, *t_h_*: delivery time for *Q*_h_, and *t*_l_: delivery time for *Q*_l_) were then determined by evaluating time constant and RBC aggregation.

**Figure 5:**
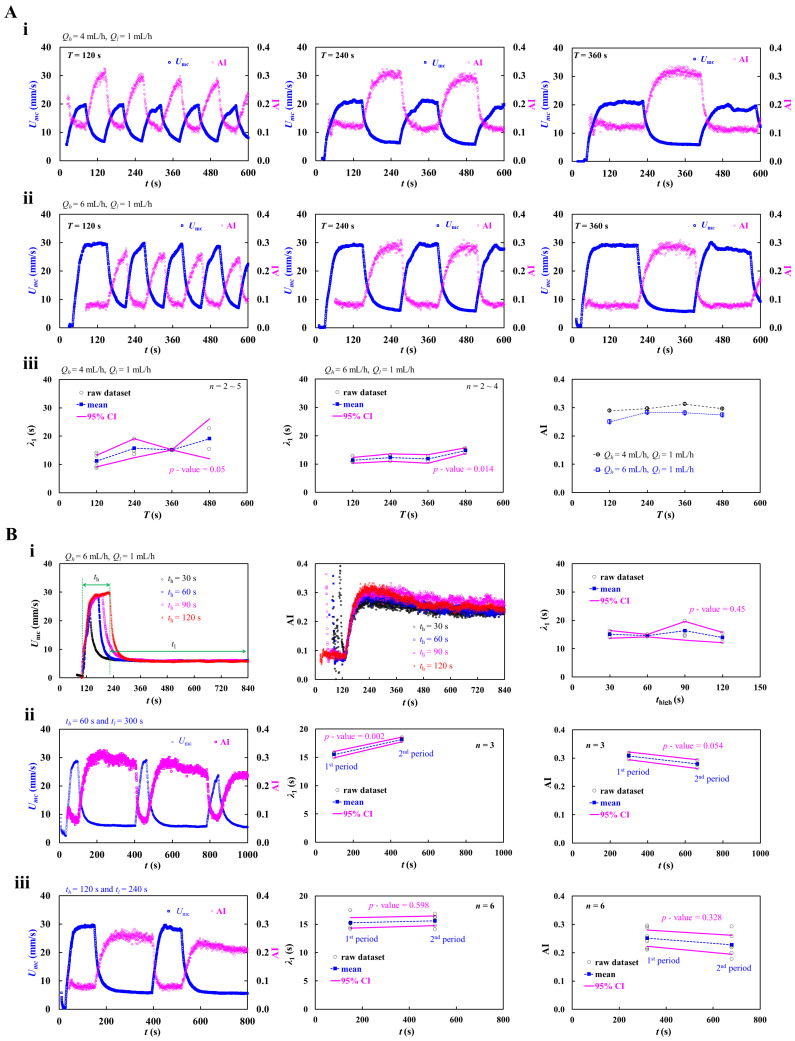
Determination of pulsatile blood flow profiles for effective measurement of time constant and RBC aggregation. (**A**) Contribution of period (*T*) and maximum flow rate (*Q*_h_) to RBC aggregation index (AI) and time constant (*λ*_1_). Period of pulsatile blood flow was set to *T* = 120~480 s. (**i**) Time-course changes in *U*_mc_ and AI with respect to *T* at *Q*_h_ = 4 mL/h. (**ii**) Time-resolved *U*_mc_ and AI as a function of *T* at *Q*_h_ = 6 mL/h. (**iii**) Quantification of time constant (*λ*_1_) and AI with respect to *T*. The left panel showed variations in *λ*_1_ as functions of *T* under *Q*_h_ = 4 mL/h. The middle panels exhibited variations in *λ*_1_ as functions of *T* at *Q*_h_ = 6 mL/h. The right panel depicted variations in AI as a function of *T* at *Q*_h_ = 4 and 6 mL/h. (**B**) Delivery duration determination of pulsatile blood flow-rate. Here, maximum and minimum flow rates were fixed at *Q*_h_ = 6 mL/h and *Q*_l_ = 1 mL/h, respectively. (**i**) Effect of delivery duration at *Q*_h_ on AI and time constant (*λ*_1_). The duration of *Q*_h_ was set to *t*_h_ = 30, 60, 90, and 120 s. The left panel showed time-resolved *U*_mc_ as a function of *t*_h_, where *t*_l_ denoted the duration at *Q*_l_. The middle panel depicted time-lapse AI with respect to *t*_h_. The right panel exhibited variations in *λ*_1_ with respect to *t*_h_. (**ii**) Variations in *λ*_1_ and AI at *t*_h_ = 60 s and *t*_l_ = 300 s. The left panel showed time-resolved *U*_mc_ and AI. The middle panel showed variations in *λ*_1_ during the first and second periods. The right panel showed variations in AI between the first and second periods. (**iii**) Variations in *λ*_1_ and AI at *t*_h_ = 120 s and *t*_l_ = 240 s. The left panel showed time-lapse *U*_mc_ and AI. The middle panel showed variations in *λ*_1_ during the first and second periods. The right panel showed variations in AI between the first and second periods.

First, the suggested method was employed to examine the effect of maximum flow rate (*Q*_h_) and overall period (*T* = *t*_h_ + *t*_l_) on time constant and aggregation index (AI). As presented in Figure 3A(ii), blood velocity remained nearly constant above *Q*_b_ = 4 mL/h, where shear rate was estimated as $\dot{\gamma }$ = 2667 s^−1^ in the main channel and $\dot{\gamma }$ = 135 s^−1^ in the test chamber, respectively. The maximum flow rate was selected as *Q*_h_ = 4, and 6 mL/h. Period of pulsatile flow rate was set to *T* = 120~480 s. As shown in Figure 5A(i), time-dependent *U*_mc_ and AI were measured at *T* = 120, 240, and 360 s under the flow rate condition (*Q*_h_ = 4 mL/h, *Q*_l_ = 1 mL/h). At *T* = 120 s, both *U*_mc_ and AI did not reach plateau values during each pulsatile flow period. When period was set to 240 s or longer, the *U*_mc_ reached a plateau value in each period. Additionally, the AI showed a stable plateau response. Under the conditions, the AI was obtained as about 0.3 at *Q*_l_ = 1 mL/h and 0.1 at *Q*_h_ = 4 mL/h, respectively. Furthermore, the *U*_mc_ decreased gradually from its plateau value when flow rate was changed from *Q*_h_ to *Q*_l_. Analytical expression of Equation (7) could be subsequently used to determine the time constant under a well-defined transient blood flow profile.

Figure 5A(ii) presented time-resolved *U*_mc_ and AI as functions of *T* under the flow-rate condition (*Q*_h_ = 6 mL/h, *Q*_l_ = 1 mL/h). When the pulsatile flow period was set to 240 s or longer, the AI reached a stable plateau value. In particular, a longer period produced a more stable AI response, indicating that sufficient delivery time at each flow rate condition was required for reliable aggregation measurement. As shown in Figure 5A(iii), variations in time constant (*λ*_1_) and AI were summarized as a function of *T* under the flow rate conditions. The left panel showed variations in *λ*_1_ as a function of *T* under the flow rate condition (*Q*_h_ = 4 mL/h, *Q*_l_ = 1 mL/h). The experiment for each period was repeated about *n* = 2~5. The results indicated that the *λ*_1_ tended to increase substantially with respect to *T* (*p*-value = 0.05). The middle panels exhibited variations in *λ*_1_ as functions of *T* under the flow rate condition (*Q*_h_ = 6 mL/h, *Q*_l_ = 1 mL/h). For each period, the experiment was repeated about *n* = 2~4. The results showed that the *λ*_1_ increased significantly with increasing *T* (*p*-value = 0.014). In addition, the *λ*_1_ exhibited more consistent values when compared with those obtained at *Q*_h_ = 4 mL/h. The right panel depicted variations in AI as a function of *T* at *Q*_max_ = 4 and 6 mL/h. Herein, the AI was calculated by averaging plateau values obtained at Q_l_ = 1 mL/h. Compared with the flow rate of *Q*_h_ = 4 mL/h, the AI exhibited lower values at Q_h_ = 6 mL/h over the tested range of *T*.

Second, although the condition of *T* = 360 s and *Q*_h_ = 6 mL/h provided stable values for the time constant and aggregation index, the 180 s delivery at *Q*_h_ = 6 mL/h resulted in considerable blood loss because measurement was not performed during the high flow rate interval. To minimize blood loss at *Q*_h_ = 6 mL/h, it was also necessary to determine the delivery duration at *Q*_h_ (*t*_h_). As shown in Figure 5B, the contribution of *t*_h_ to both time constant and AI was quantitatively examined under the flow rate condition (*Q*_h_ = 6 mL/h, *Q*_l_ = 1 mL/h). As shown in Figure 5B(i), the effect of delivery duration at *Q*_h_ on both AI and time constant (*λ*_1_) was quantitatively examined. The delivery duration of *Q*_h_ was set to *t*_h_ = 30, 60, 90, and 120 s. The left panel showed time-resolved *U*_mc_ as a function of *t*_h_, where *t*_l_ denoted the delivery duration at *Q*_l_. Except for the condition of *t*_h_ = 30 s, the *U*_mc_ reached a plateau value during the high flow rate interval. The middle panel presented time-lapse AI with respect to *t*_h_. The results indicated that a longer *t*_h_ contributed to increasing AI. The right panel exhibited variations in *λ*_1_ as a function of *t*_h_. Unlike AI, the *λ*_1_ did not show a substantial difference with respect to *t*_h_ (*p*-value = 0.45).

Based on the results, the delivery duration at *Q*_h_ was set to *t*_h_ = 60 s or longer. As shown in Figure 5B(ii), variations in *λ*_1_ and AI were obtained at a specific delivery condition (i.e., *t*_h_ = 60 s, *t*_l_ = 300 s, and *T* = 360 s). The left panel showed time-resolved *U*_mc_ and AI. As the plateau interval at *Q*_h_ was short, the AI did not show a plateau value. However, the AI exhibited a plateau value at longer delivery duration at *Q*_l_.

The middle panel showed variations in *λ*_1_ during the first and second periods. For each period, measurements were repeated about *n* = 3. The results showed that the *λ*_1_ increased remarkedly after an elapsed period (*p*-value = 0.002). The right panel presented variations in AI between the first and second periods. In contrast to *λ*_1_, the AI decreased after an elapsed period, with marginal statistical significance (*p*-value = 0.054). Because the *λ*_1_ increased remarkedly at *t*_h_ = 60 s, the delivery duration at Q_h_ was extended to *t*_h_ = 120 s. The delivery duration at *Q*_l_ was then adjusted to *t*_l_ = 240 s (i.e., *T* = 360 s). As shown in Figure 5B(iii), *λ*_1_ and AI were measured under this optimized delivery condition (i.e., *t*_h_ = 120 s, *t*_l_ = 240 s). The left panel showed time-lapse *U*_mc_ and AI. Both *U*_mc_ and AI showed stable plateau values at Q_h_ and *Q*_l_. The middle panel presented variations in *λ*_1_ during the first and second periods. For each period, measurement was repeated about *n* = 6. The results indicated that the *λ*_1_ remained nearly constant after an elapsed period (*p*-value = 0.598). The right panel showed variations in AI between the first and second periods. According to the results, AI showed a decreasing tendency after an elapsed period and the difference was not statistically significant (*p*-value = 0.328).

From the experimental investigation, the pulsatile blood flow profile was optimized to reliably measure both the time constant (λ_1_) and the RBC aggregation index (AI) while reducing unnecessary blood consumption. The final condition was set to *Q*_h_ = 6 mL/h, *Q*_l_ = 1 mL/h, *t*_h_ = 120 s, and *t*_l_ = 240 s. Under this delivery condition, *U*_mc_ and AI reached stable plateau values, and λ_1_ remained reproducible across repeated periods, confirming that the optimized profile could provide a robust condition for simultaneous assessment of transient blood flow response and RBC aggregation.

### 3.5. Contribution of Air Compliance to Time Constant

Because the air cavity secured inside the syringe acts as a compliance element [42], it can dampen flow fluctuations while increasing the transient response time of the microfluidic system [57,65]. Consequently, the compliance-induced delay may affect the plateau value of AI by altering the time required to establish a stable low-shear condition for RBC aggregation measurement.

**Figure 6:**
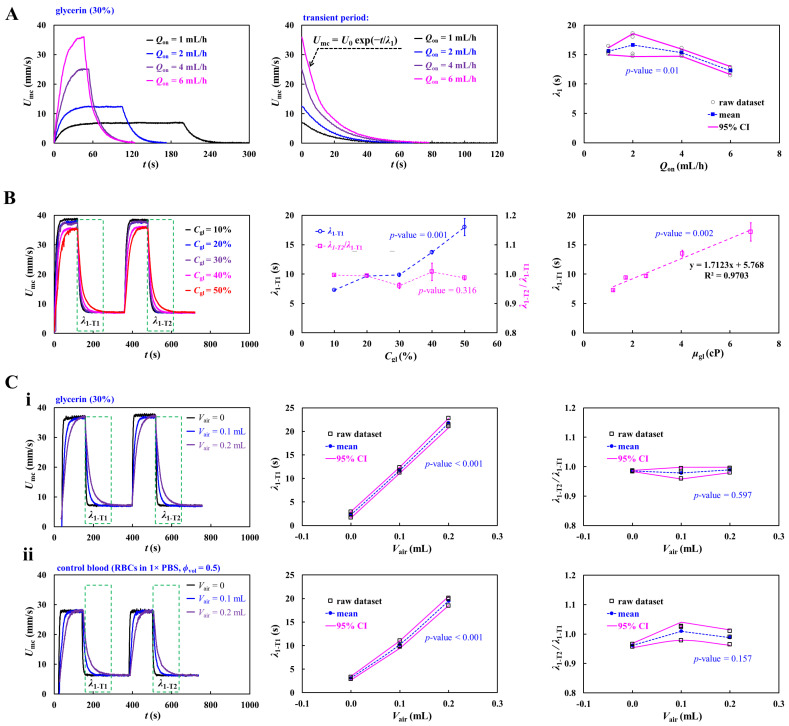
Contribution of air cavity secured in syringe and flow rate to time constant and RBC aggregation index. (**A**) Effect of flow rate on time constant. The left panel showed time-course *U*_mc_ with respect to *Q*_on_. The middle panel depicted time-resolved *U*_mc_ during the transient interval. The right panel exhibited variations in *λ*_1_ with respect to *Q*_on_. (**B**) Variations in time constant as a function of glycerin concentration (*C*_gl_) during the first and second periods. The left panel showed time-resolved *U*_mc_ as a function of *C*_gl_ = 10~50%. The middle panel exhibited variations in *λ*_1-T1_ and *λ*_1-T2_/*λ*_1-T1_ with respect to *C*_gl_. The right panel depicted linear correlation between time constant (*λ*_1-T1_) and viscosity (*μ*_gl_). (**C**) Contribution of air cavity (*V*_air_) inside the syringe to time constant for glycerin and blood sample. (**i**) Variations in time constant for glycerin (*C*_gl_ = 30%) with respect to *V*_air_. The left panel showed time-lapse *U*_mc_ with respect to *V*_air_. The middle panel exhibited variations in *λ*_1-T1_ with respect to *V*_air_. The right panel showed variations in *λ*_1-T2_/*λ*_1-T1_ with respect to *V*_air_. (**ii**) Variations in time constant for test blood (normal RBCs into 1× PBS, *ϕ*_vol_ = 0.5) with respect to *V*_air_. The left panel showed time-lapse *U*_mc_ with respect to *V*_air_. The middle panel exhibited variations in *λ*_1-T1_ with respect to *V*_air_. The right panel showed variations in *λ*_1-T2_/*λ*_1-T1_ with respect to *V*_air_ = 0.1 mL. The left panel showed time-lapse *U*_mc_ as a function of *C*_gl_ = 10~50%. Herein, *λ*_1-T1_ and *λ*_1-T2_ denoted time constants obtained during the first and second periods, respectively. The middle panel presented variations in *λ*_1-T1_ and *λ*_1-T2_/*λ*_1-T1_ with respect to *C*_gl_. The *λ*_1-T1_ increased significantly with increasing *C*_gl_ (*p*-value = 0.001). In contrast, *λ*_1-T2_/*λ*_1-T1_ did not show significant dependence on C_gl_ (*p*-value = 0.316), indicating that the time constant remained consistent between two periods. To examine the correlation between time constant (*λ*_1-T1_) and viscosity (*μ*_gl_) [66], *λ*_1-T1_ was plotted against *μ*_gl_ in the right panel. Linear regression analysis showed a strong proportional relationship between the time constant and viscosity, expressed as *λ*_1-T1_ = 1.7123 *μ*_gl_ + 5.768 (R^2^ = 0.9703, and *p*-value = 0.002). The results indicated that the time constant could be effectively used to monitor viscosity variations.

As shown in Figure 6A, the effect of flow rate on time constant was examined by measuring *λ*_1_ as a function of plateau flow rate. Instead of test blood, glycerin (*C*_gl_ = 30%) was used as the test fluid. The air cavity above glycerin inside the syringe was set to *V*_air_ = 0.1 mL.

Fluid flow was abruptly stopped from a plateau value of *Q*_on_ = 1, 2, 4, and 6 mL/h. The left panel showed time-dependent *U*_mc_ as a function of *Q*_on_. In the middle panel, the *U*_mc_ was replotted from the onset of transient flow. The results showed that the *U*_mc_ decreased more slowly at higher flow rates. Based on single exponential model, the transient velocity was fitted using $U_{mc}(t)=U_{0}exp(-\frac{t}{\gamma_{1}})$ for estimating the time constant (*λ*_1_). The right panel exhibited variations in *λ*_1_ with respect to *Q*_on_. The *λ*_1_ did not show a substantial difference below *Q*_on_ = 4 mL/h. However, when Q_on_ increased from 4 mL/h to 6 mL/h, the *λ*_1_ increased remarkedly (*p*-value = 0.01). This trend for the glycerin solution was similar to that observed for the control blood, as shown in Figure 3A(i).

As shown in Figure 6B, the time constant was evaluated as a function of glycerin concentration (*C*_gl_) during the first and second periods. Herein, glycerin solution was infused under the optimized pulsatile flow profile, and the air cavity inside a syringe was fixed at *V*_air_.

As shown in Figure 6C, time constants for glycerin and control blood were measured as a function of the air cavity (*V*_air_). Both fluids were infused under the optimized flow profile. The air cavity inside the syringe was set to *V*_air_ = 0, 0.1, and 0.2 mL. Figure 6C(i) depicted the time constant for glycerin (*C*_gl_ = 30%) as a function of *V*_air_. The left panel presented time-lapse *U*_mc_ with respect to *V*_air_. By analyzing transient flow response during the first and second periods, two time constants (*λ*_1-T1_, *λ*_1-T2_) were calculated for each period. The middle panel exhibited variations in *λ*_1-T1_ with respect to *V*_air_. As expected, the *λ*_1-T1_ increased markedly with respect to *V*_air_ (*p*-value < 0.001), confirming the strong influence of air compliance on the transient flow response. The right panel presented variations in *λ*_1-T2_/*λ*_1-T1_ with respect to *V*_air_. From the results, the ratio did not show significant dependence on *V*_air_ (*p*-value = 0.597), indicating that the time constant remained consistent between the two periods. As shown in Figure 6C(ii), the time constant for test blood (normal RBCs into 1× PBS, *ϕ*_vol_ = 0.5) was measured as a function of *V*_air_. The left panel showed time-lapse *U*_mc_ with respect to *V*_air_. The middle panel exhibited variations in *λ*_1-T1_ with respect to *V*_air_. The *λ*_1-T1_ increased significantly with the increasing air cavity (*p*-value < 0.001). The right panel showed variations in *λ*_1-T2_/*λ*_1-T1_ with respect to *V*_air_. The results indicated that the ratio did not show significant difference with respect to V_air_ (*p*-value = 0.157), suggesting that the time constant remained reproducible between the two periods.

From the experimental investigation, the air cavity (*V*_air_) significantly increased the time constant (*λ*_1-T1_) in both the glycerin solution and control blood, confirming that syringe air compliance strongly delayed the transient flow response. However, *λ*_1-T2_/*λ*_1-T1_ remained nearly unchanged with respect to *V*_air_, indicating good reproducibility between repeated periods.

### 3.6. Monitoring Variation in Blood During Continuous Blood Infusion

Because RBC aggregation accelerates sedimentation in syringes [27,56,64,67,68], the hematocrit of delivered blood could vary continuously during blood infusion. As the final demonstration, the proposed method was applied to detect these changes in supplied blood by simultaneously monitoring two parameters, including time constant and RBC aggregation index. Herein, for consistent measurement, air cavity inside the syringe was fixed at *V*_air_ = 0.1 mL. Test blood (normal RBCs into dextran solution) was then infused into a microfluidic device under the optimized pulsatile flow profile.

**Figure 7:**
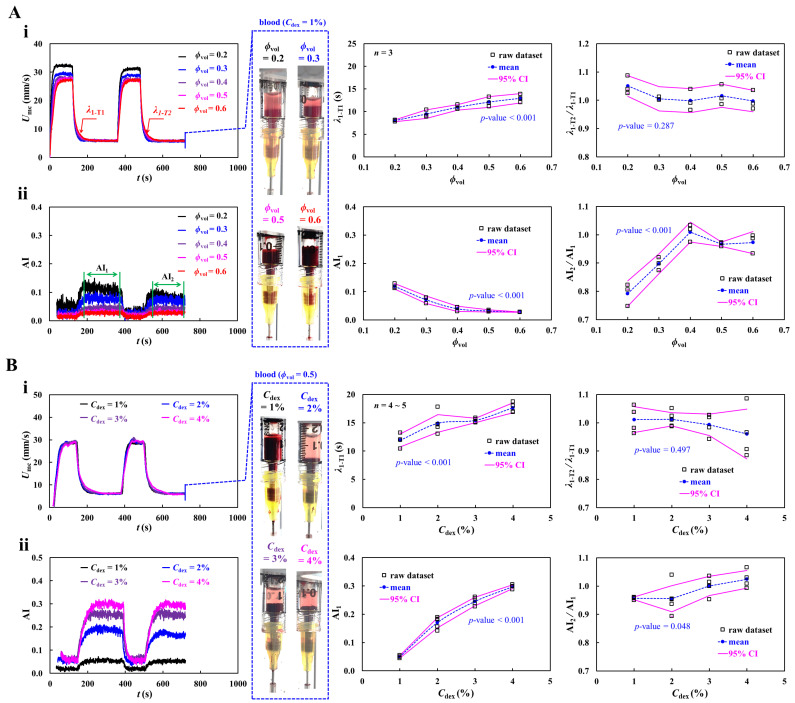
Monitoring alternating blood properties during continuous infusion with a syringe pump. (**A**) Contribution of hematocrit to time constant and AI. (**i**) Variations in time constant as a function of hematocrit during the first and second periods. The first panel showed time-lapse *U*_mc_ as a function of hematocrit (*ϕ*_vol_). The second panel depicted snapshots of the syringe captured at the completion of blood delivery. The third panel exhibited variations in *λ*_1-T1_ as a function of *ϕ*_vol_. The last panel showed variations in *λ*_1-T2_/*λ*_1-T1_ with respect to *ϕ*_vol_. (**ii**) Variations in RBC aggregation index as a function of hematocrit during the first and second periods. The left panel showed time-dependent AI as a function of *ϕ*_vol_. The middle panel represented variations in AI_1_ with respect to *ϕ*_vol_. The right panel depicted variations in AI_2_/AI_1_ as a function of *ϕ*_vol_. (**B**) Contribution of dextran concentration to time constant and AI. (**i**) Variations in time constant as a function of dextran concentration during the first and second periods. The first panel showed time-lapse *U*_mc_ as a function of dextran concentration (*C*_dex_). The second panel depicted snapshots of the syringe captured at the completion of blood delivery. The third panel exhibited variations in *λ*_1-T1_ as a function of *C*_dex_. The last panel showed variations in *λ*_1-T2_/*λ*_1-T1_ with respect to *ϕ*_vol_. (**ii**) Variations in RBC aggregation index as a function of dextran concentration during the first and second periods. The left panel showed time-dependent AI as a function of *C*_dex_. The middle panel represented variations in AI_1_ with respect to *C*_dex_. The right panel depicted variations in AI_2_/AI_1_ as a function of *C*_dex_.

First, as shown in Figure 7A, to examine the effect of hematocrit on both the time constant and RBC aggregation index, time constant (λ_1_) and AI were measured by varying hematocrit (*ϕ*_vol_). Herein, the hematocrit of test blood was adjusted to *ϕ*_vol_ = 0.2, 0.3, 0.4, 0.5, and 0.6 by suspending normal RBCs into a dextran solution of *C*_dex_ = 1%. Figure 7A(i) showed variations in time constant as a function of hematocrit during the first and second periods. The first panel presented time-lapse *U*_mc_ as a function of *ϕ*_vol_ = 0.2~0.6. Herein, *λ*_1-T1_ and *λ*_1-T2_ were obtained by analyzing the transient flow response during the first and second periods. The second panel depicted snapshots of the syringe captured at the completion of blood delivery. From the results, the RBC-depleted layer increased significantly at lower hematocrit. At *ϕ*_vol_ = 0.6, the RBC-depleted layer was not detected clearly. The third panel exhibited variations in *λ*_1-T1_ as a function of *ϕ*_vol_. For each hematocrit, the measurement was repeated three times (*n* = 3). As expected, hematocrit contributed to significantly increasing *λ*_1-T1_ (*p*-value < 0.001), which was consistent with previous hemorheological studies showing that hematocrit was a major determinant of whole-blood viscosity and flow resistance [69,70,71]. Therefore, a higher hematocrit could prolong the transient flow response in a compliant microfluidic system. The last panel showed variations in *λ*_1-T2_/*λ*_1-T1_ with respect to *ϕ*_vol_. The ratio did not show substantial dependence on hematocrit (*p*-value = 0.287), indicating that time constant remained consistent between two periods. As shown in Figure 7A(ii), RBC aggregation index (AI) was measured as a function of hematocrit during the first and second periods. The left panel showed time-dependent AI as a function of *ϕ*_vol_. Herein, AI_1_ and AI_2_ were determined by averaging the plateau values at *Q*_l_ during the first and second periods, respectively. The middle panel represented variations in AI_1_ with respect to *ϕ*_vol_. The AI_1_ decreased markedly with increasing hematocrit (*p*-value < 0.001). The right panel depicted variations in AI_2_/AI_1_ as a function of *ϕ*_vol_. The ratio increased significantly up to *ϕ*_vol_ = 0.4 (*p*-value < 0.001), whereas no substantial difference was observed from *ϕ*_vol_ = 0.4 to *ϕ*_vol_ = 0.6. From the results, RBC sedimentation occurred during blood delivery and significantly reduced AI, particularly at lower hematocrit (*ϕ*_vol_ = 0.2~0.4).

Second, to examine the contribution of dextran concentration to time constant and AI, as shown in Figure 7B, time constant and AI were probed as a function of dextran concentration. Herein, test blood (*ϕ*_vol_ = 0.5) was prepared by adding normal RBCs into dextran solution (*C*_dex_ = 1~4%). Figure 7B(i) presented variations in time constant as a function of dextran concentration during the first and second periods. The first panel showed time-lapse *U*_mc_ as a function of *C*_dex_. The second panel depicted snapshots of the syringe captured at the completion of blood delivery. At *C*_dex_ = 1%, RBC-depleted layer was much smaller than those observed at a higher concentration of dextran solution (*C*_dex_ = 2~4%). The third panel exhibited variations in *λ*_1-T1_ as a function of *C*_dex_. The results indicated the dextran concentration significantly increased the time constant (*p*-value < 0.001). The last panel showed variations in *λ*_1-T2_/*λ*_1-T1_ with respect to *C*_dex_. Although the ratio tended to decrease from *C*_dex_ = 2% to *C*_dex_ = 4%, the change was not statistically significant (*p*-value = 0.497). Figure 7B(ii) exhibited variations in AI as a function of dextran concentration during the first and second periods. The left panel showed time-dependent AI as a function of *C*_dex_. The results indicated that the AI increased remarkably at a higher concentration of dextran solution. The middle panel represented variations in AI_1_ with respect to *C*_dex_. The AI_1_ increased significantly with increasing dextran concentration (*p*-value < 0.001). The right panel depicted variations in AI_2_/AI_1_ as a function of *C*_dex_. The ratio showed a significant dependence on dextran concentration (*p*-value < 0.048), indicating that the AI varied substantially between the two periods.

From the experimental investigation, the proposed method enabled effective monitoring of time-dependent changes in blood properties during continuous infusion. By simultaneously measuring the time constant (λ_1_) and RBC aggregation index (AI), the method detected infusion-induced variations in the delivered blood. Specifically, λ_1_ reflected changes in flow resistance and viscosity-related properties, whereas AI quantified variations in RBC aggregation. These results demonstrated that the proposed approach could provide a practical and sensitive platform for real-time assessment of blood property changes during continuous delivery.

The proposed method has several limitations for practical applications. First, it was validated mainly under controlled laboratory conditions using prepared blood samples with defined hematocrit and dextran concentrations. Second, the current platform still depends on external syringe-pump control, which may limit its immediate clinical translation. Further validation using fresh clinical blood samples and integration with a portable fluid-delivery system (i.e., air pressure controller) will be necessary to improve its robustness and practical applicability. Third, the effects of BSA treatment, PDMS hydrophobic recovery, chip age after plasma bonding, and device reuse were not evaluated quantitatively. Thus, controlled surface-condition studies and transition toward thermoplastic-based disposable microfluidic chips for practical and standardized hemorheological testing will be considered in future work. Lastly, future studies should evaluate microfluidic chip operation time (i.e., adhesion, clogging, leakage, bonding stability) for practical hemorheological testing.

## 4. Conclusions

In this study, the proposed microfluidic method enabled simultaneous evaluation of viscosity-related transient flow behavior and RBC aggregation under continuous pulsatile blood delivery. An optimized pulsatile-flow profile was applied by periodically switching the flow rate between high flow rate (*Q*_h_ = 6 mL/h for 2 min) and low flow rate (*Q*_l_ = 1 mL/h for 4 min). The air cavity inside the syringe was fixed at *V*_air_ = 0.1 mL. The time constant (λ_1_) showed a strong correlation with fluid viscosity, supporting its use as a sensitive indicator of flow resistance and viscosity-related changes. The proposed aggregation index (AI) exhibited consistent trends with hematocrit and dextran concentration when compared with two previous indices (AI_p1_ and AI_p2_), while providing improved temporal stability under continuous-flow conditions. By optimizing the pulsatile-flow profile, reliable measurements of both λ_1_ and AI were achieved with reduced blood consumption. The air cavity inside the syringe acted as a compliance element and significantly influenced the time constant, confirming the importance of controlling system compliance. Finally, the method successfully detected time-dependent changes in delivered blood during continuous infusion, demonstrating its potential as a practical and sensitive platform for real-time monitoring of hemorheological variations.

## Data Availability

The original contributions presented in this study are included in the article. Further inquiries can be directed to the corresponding author.
